# Comparative study of different extendable intramedullary rods combined with surgery in the treatment of congenital pseudarthrosis of the tibia

**DOI:** 10.1186/s13023-024-03202-0

**Published:** 2024-05-21

**Authors:** Yaoxi Liu, Kun Liu, Qian Tan, Ge Yang, Yiyong Huang, Guanghui Zhu, Jiangyan Wu, Haibo Mei

**Affiliations:** Department of pediatric orthopedics, The Affiliated Children’s Hospital Of Xiangya School of Medicine,Central South University (Hunan children’s hospital), Hunan Provincial Key Laboratory of Pediatric Orthopedics, The school of pediatrics,University of South China, 86 Ziyuan Road, Changsha City, Hunan Province 410007 People’s Republic of China

**Keywords:** Children, Extendable intramedullary rod, Contrastive study

## Abstract

**Background:**

When using traditional extensible intramedullary rods to treat congenital pseudarthrosis of the tibia (CPT), there were cases of re-fracture and internal fixation fracture. Therefore, the authors propose a research hypothesis that a thicker distal extensible intramedullary rod can better protect the tibia and reduce the incidence of refracture

**Purpose:**

To investigate the clinical efficacy of new and traditional extensible intramedullary rods in the treatment of CPT in children

**Methods:**

From January 2017 to December 2021, the clinical data of 49 children with CPT who were treated with traditional extensible intramedullary rod combined surgery (group A) and new extensible intramedullary rod combined surgery (group B) in our hospital were collected. Inclusive criteria: ① Crawford type IV CPT children; ② The operation was performed by the same team. Exclusion criteria: patients with multiple tibial angulation. During follow-up, the initial healing, proximal tibial valgus, tibial length, ankle valgus, refracture and intramedullary rod displacement of CPT children in the two groups were evaluated

**Results:**

It was a retrospective investigation. In group A, 26 cases met the inclusion criteria, 24 cases achieved primary healing, with an primary healing rate of 92%, including 1 case of nonunion due to osteomyelitis complications after surgery, and 1 case of delayed healing, with an average healing time of 4.7 ± 0.8 months. 17 cases (68%) had unequal tibia length, with an average difference of 1.6 ± 0.8 cm. Ankle valgus occurred in 10 cases (40%) with an average of 14.4°±4.8°; Proximal tibial valgus occurred in 6 cases (24%) with an average of 7 °± 1.8 °. 20 cases (80%) had tip of the rod migration.10 cases (40%) had re-fracture; The average follow-up time was 2.4 ± 0.4 years. In group B, 22 patients achieved primary healing, and the primary healing rate was 95%, including 1 case with delayed healing. The average healing time was 4.7 ± 1.7months. 14 cases (61%) had unequal tibia length, with an average difference of 1 ± 0.5 cm. Ankle valgus occurred in 4 cases (17%) with an average of 12.3 °±4.9°; The proximal tibia valgus occurred in 9 cases (39%), with an average of 7.7 °±2.5 °. 14 cases (61%) had new type of intramedullary rod displacement. 3 cases (13%) had re-fracture; The average follow-up time was 2.3 ± 0.6years

**Conclusion:**

Compared with the traditional extended intramedullary rod combined operation, the new type of extended intramedullary rod combined operation has a lower incidence of re-fracture after CPT, but it still needs to be verified by large sample and multi-center research

**Supplementary Information:**

The online version contains supplementary material available at 10.1186/s13023-024-03202-0.

## Introduction

**Fig. 1:**
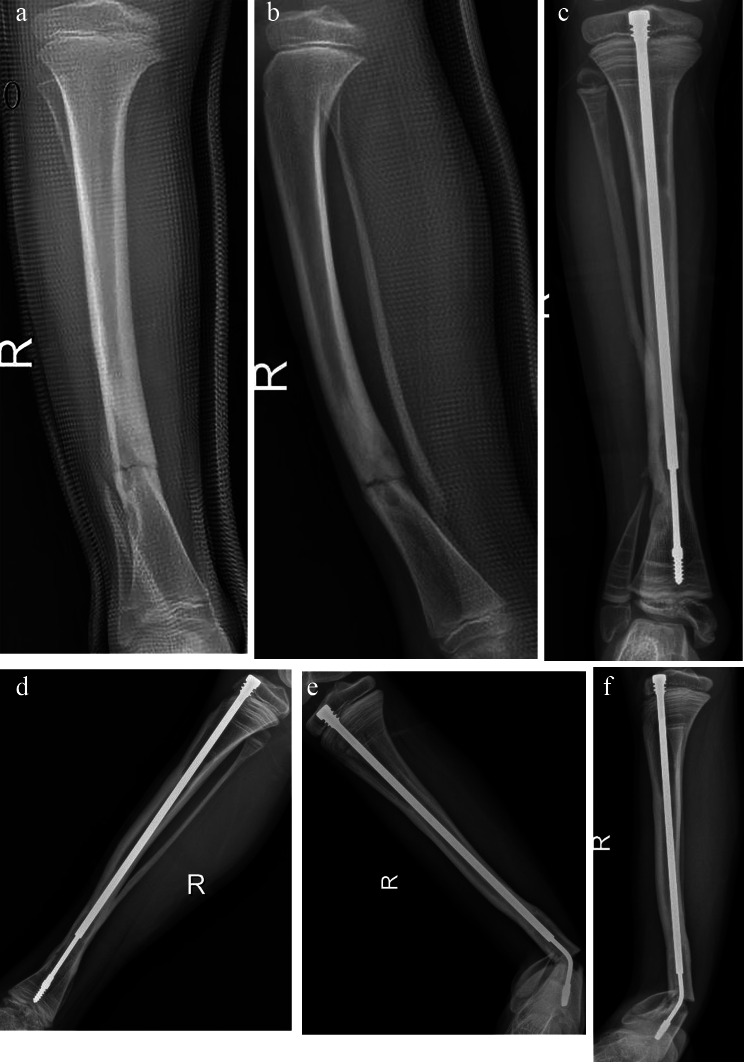
**A** and **B**: X-rays of the tibia of a 9-year-old male child with CPT before operation; **C** and **D**: Anteroposterior and lateral radiographs in two years and three months after CPT showed distal displacement of the intramedullary rod. **E** and **F**: Anteroposterior and lateral radiographs at 2 years and 10 months after CPT showed re-fracture with bent inner core

Congenital pseudarthrosis of the tibia (CPT) is a rare and refractory disease in pediatric orthopedics [1–4], which often requires multiple operations, and the family members of patients often suffer from great economic and psychological pressure. With the development of surgical technology, although the primary union rate of tibial pseudarthrosis is significantly improved [2], the risk of amputation has not been completely eliminated. The therapeutic objective of CPT is to achieve the healing of tibial pseudarthrosis, minimize the occurrence of postoperative complications, and improve the quality of life of children. After the initial healing of CPT, there are ankle valgus, unequal length of tibia, proximal tibia valgus, re-fracture and other complications. Among them, re-fracture is the most serious complication, because it may lead to the formation of new tibial pseudarthrosis. The incidence of re-fracture reported in the literature is 11 − 68%, which may have a small cross-sectional area with the healing area of tibial pseudarthrosis, accompanied by fibular pseudarthrosis, abnormal tibial axis, poor compliance of wearing brace, no intramedullary fixation, biological factors related to hamartoma recurrence [4]. If the cast immobilization does not heal after re-fracture, surgery may be required. The intramedullary rod may have the effect of preventing re-fracture. In the past, Williams rods were often used in surgery, but the traditional intramedullary fixation through the ankle would affect the ankle function. The expandable intramedullary rod does not pass through the ankle, so it does not affect the function of the ankle. However, the traditional extensible intramedullary rod has some shortcomings, such as small diameter of the inner core of the intramedullary rod, easy bending [5], fracture (Fig. 1), and cannot prevent the distal tibia from re-fracture [6]. In 1990, Fern E et al. [7] reported that 5 patients with CPT were treated with expandable intramedullary rods, and one of them suffered from bending and re-fracture of the core of the expandable intramedullary rods. In our hospital, when using traditional extensible intramedullary rods to treat CPT, there were cases of re-fracture and inner core-fracture. Therefore, the authors propose a research hypothesis that a thicker distal extensible intramedullary rod can better protect the tibia and reduce the incidence of refracture. The authors improved the intramedullary rod and designed a new type of expandable intramedullary rod for patients, aiming at better preventing the occurrence of re-fracture. The purpose of this paper was to investigate the clinical effect of the combination of new and traditional expandable intramedullary rods in the treatment of CPT.

## Patients and methods

**Fig. 2:**
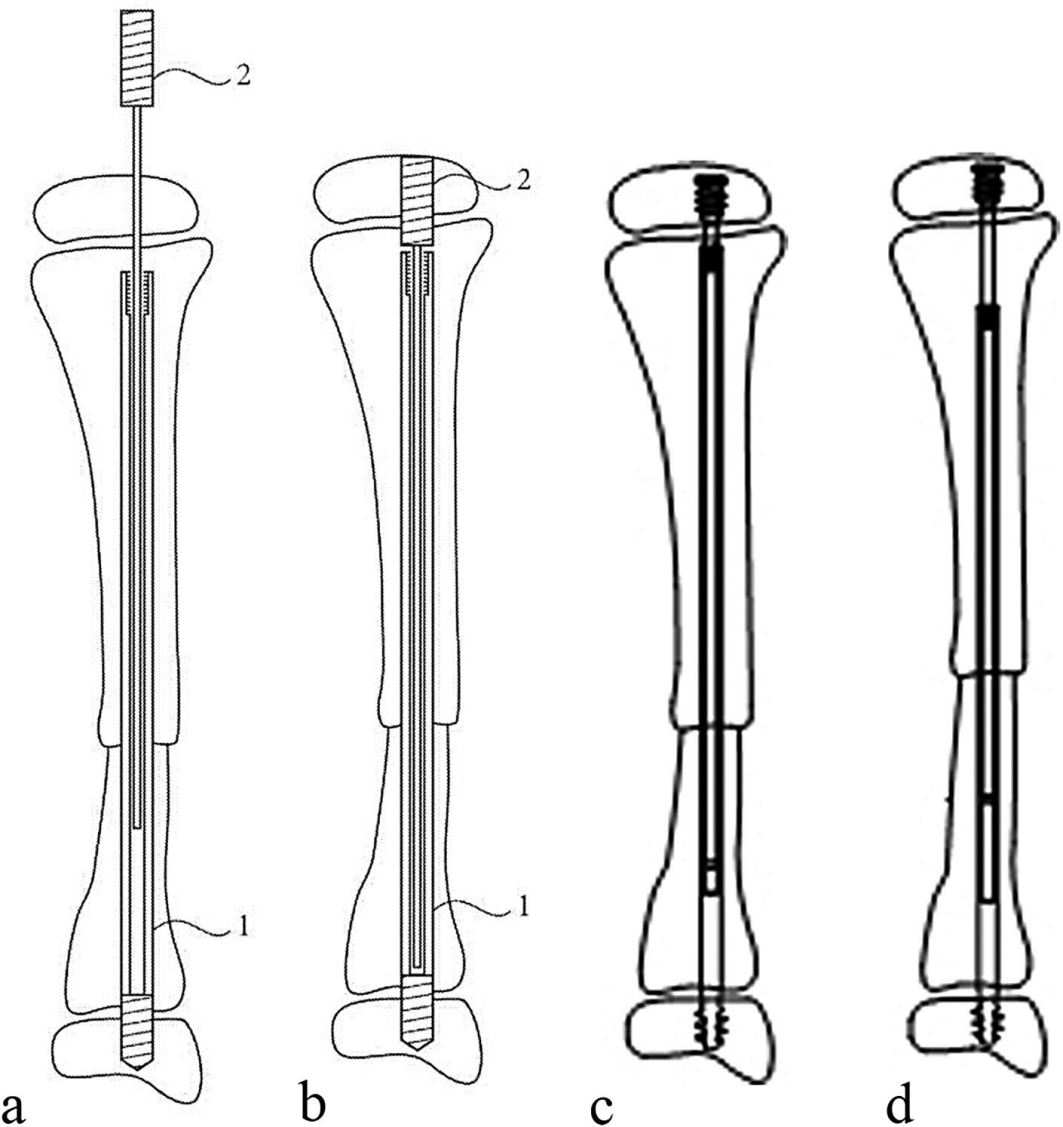
Schematic diagram of new extensible intramedullary rod Note 1: external sleeve; 2 Inner core. (The outer sleeve rod is composed of a threaded near end and a sleeve part connecting the tip head. The core rod is composed of a threaded flat end and a core rod connecting the flat end. The mandrel is sleeved in the hollow sleeve tube body, and the top end of the flat end of the inner core is provided with an inner hexagon hole; The inner side of the opening end of the outer sleeve tube body is provided with an internal thread section, and the outer sleeve rod is fixedly connected with the internal thread section with a mounting rod during installation. The threaded tip is fixed in the distal tibial epiphysis.)

A total of 49 children with CPT who were treated in our hospital from January 2017 to December 2021 with traditional extensible intramedullary rod combined surgery (Group A) and new extensible intramedullary rod combined surgery (Group B) (Fig. 2) [8] were collected. Inclusion criteria: ① Crawford type IV CPT children, ② The operation was completed by the same team. Exclusion criteria: patients with multiple tibial angulations. This study has been reviewed and approved by the Hospital Ethics Committee (HCHLL - 2019 ‐ 37). After discharge, patients were followed up at the outpatient clinic once two months to evaluate the initial healing of the tibial pseudarthrosis, and the occurrence of complications such as tibial unequal length, proximal tibial valgus, ankle valgus, and refracture [6]. The RUST scoring standard [9] was used to evaluate the healing, and the image archiving and communication system was used to measure the tibial length, proximal tibial valgus, and ankle valgus. Statistical SPSSl8.0 software was used to statistically analyze the initial healing rate of tibial pseudarthrosis, re-fracture rate, incidence rate of ankle valgus, incidence rate of tibial valgus, incidence rate of tibial unequal length, and rate of intramedullary rod displacement between the two groups. Chi square test was used for statistical method, Statistical significance was considered at *P* less than 0.05.

## Results

All patients were followed up. There were 20 males and 6 females in group A; 14 cases on the left and 12 cases on the right; The average age at the time of operation was 51.8 ± 31.3 months. 9 cases were associated with proximal tibial dysplasia [10], and 22 cases were associated with neurofibromatosis type 1 (NF1). There were 16 males and 7 females in Group B:; 12 on the left and 11 on the right; The average age at the time of operation was 34.5 ± 11.6 months. 4 cases were associated with proximal tibial dysplasia and 19 cases with NF1.

26 cases met the inclusion criteria in group A, 24 cases achieved primary healing, with an primary union rate of 92%, including 1 case did not achieve union due to osteomyelitis complications after surgery, and 1 case of delayed healing, with an average healing time of 4.7 ± 0.8 months. 17 cases (68%) had unequal tibia length, with an average difference of 1.6 ± 0.8 cm. Ankle valgus occurred in 10 cases (40%) with an average of 14.4°±4.8°; Proximal tibial valgus occurred in 6 cases (24%) with an average of 7 °± 1.8 °. 20 cases (80%) had tip of the rod migration. 10 cases (40%) had re-fracture; The average follow-up time was 2.4 ± 0.4 years.

**Fig. 3:**
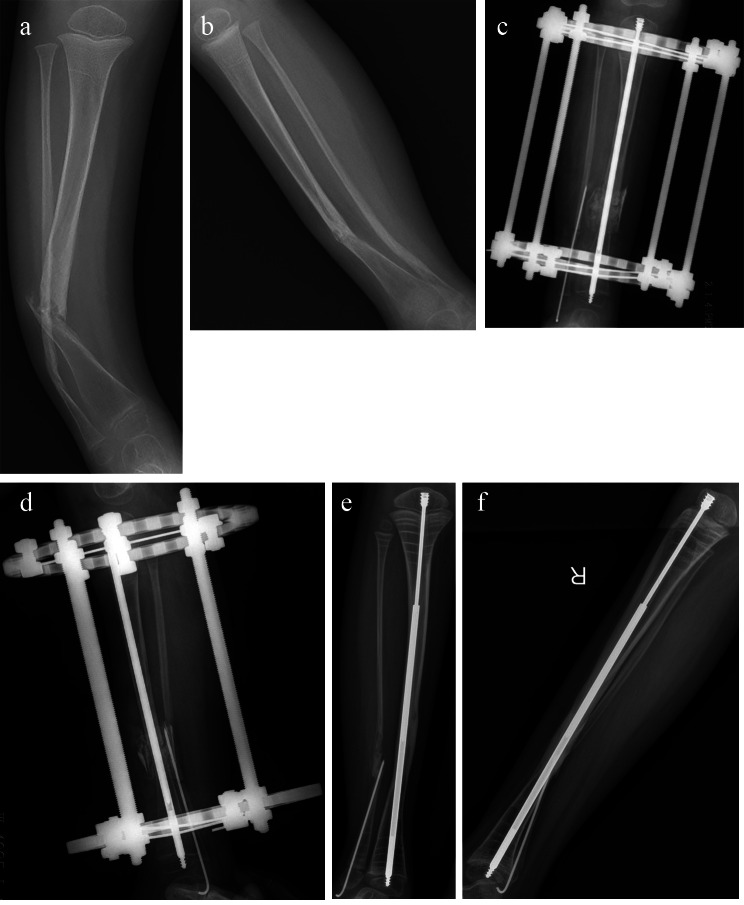
Female CPT children aged 3 years, **A** and **B**: Anteroposterior and lateral radiographs before operation; **C**, **D**: Three days after operation, X ray of tibia showed that the new type of children’s extensible intramedullary rod was in good position; **E**. **F**: Two years and three months after the operation, X ray of the tibia showed good healing at the tibial pseudarthrosis, no proximal tibial valgus and no intramedullary rod displacement

22 patients achieved primary healing in group B, and the primary healing rate was 95%, including 1 case with delayed healing. The average healing time was 4.7 ± 1.7months. 14 cases (61%) had unequal tibia length, with an average difference of 1 ± 0.5 cm. Ankle valgus occurred in 4 cases (17%) with an average of 12.3 °±4.9°; The proximal tibia valgus occurred in 9 cases (39%), with an average of 7.7 °±2.5 °. 14 cases (61%) had tip of the rod migration. 3 cases (13%) had re-fracture; The average follow-up time was 2.3 ± 0.6years. (Table 1) Fig. 3. (typical cases)

**Table 1:** Initial healing and complications between groups A and B

Group	Union rate	Ankle Valgus	PTV	TLD	Refracture	Intramedullary rod displacement
**Group A**	92%	40%(10/25)	24%(6/25)	68%(17/25)	40%(10/25)	80%(20/25)
**Group B**	95%	17%(4/23)	39%(39%)	61%(14/23)	13%(3/23)	61%(14/23)
***P***	> 0.05	> 0.05	> 0.05	> 0.05	0.036	> 0.05

*Note*: TLD: tibial length discrepancy, PTV: proximal tibial valgus

## Discussion

The role of intramedullary rods, as a central internal fixation device, plays an important role in the healing and prevention of re-fracture of CPT. Ordinary intramedullary rods may shift or become shorter with the growth of tibia. The traditional extensible intramedullary rod has a small inner core, which cannot prevent the occurrence of re-fracture. However, the diameter of the distal part of the new extensible intramedullary rod is relatively large, it may be able to better maintain the healing of the tibial pseudarthrosis and prevent the tibial re-fracture. Dustin Singer et al. [11] reported that 34 children with CPT were treated with intramedullary rods combined with autologous bone grafting, with an average follow-up of 11.9 years. 13 cases had re-fracture, of which 3 cases were not achieved union after surgical treatment. This scholar believes that intramedullary rods can maintain the union of tibial pseudarthrosis for a long time. Johnston et al. [12] pointed out that the use of intramedullary rods can promote union of tibial pseudarthrosis, maintain a good tibial mechanical axis and prevent re-fracture. Dobbs et al. [13] reported the long-term results of 31 cases treated with intramedullary rods. Among them, 12 cases suffered from re-fracture, and 3 cases recovered ankle motion by taking out the intramedullary rods, but all of them suffered from re-fracture soon. The scholar supported the idea of retaining intramedullary rods in the tibia. The meta-analysis results of Kesireddy et al. [14] showed that the incidence of refracture was 35% when CPT was treated with Ilizarov fixation alone, while the incidence of refracture was reduced to 16% when intramedullary rod was used together. In addition, Anderson et al. [15] suggested that the intramedullary rods should be retained until the time of skeletal maturity. Vanderstappen et al. [16] reported that 12 patients with CPT were treated with external fixation. The initial healing rate was 83%, and 6 patients suffered from re-fracture. The scholar believed that the bone biomechanical strength of the healing area of the tibial pseudarthrosis was not enough, so he recommended that patients use internal fixation devices to prevent re-fracture.

The authors also believe that if the intramedullary rod is in good position, it is recommended to retain the intramedullary rod in the tibia for a long time to prevent re-fracture. As a matter of fact, the site of re-fracture in children with CPT after primary union is usually in the middle and lower 1/3 of the distal tibia, in this study, 10 cases of group A had refracture, of which 7 cases occurred in the middle and lower 1/3 of the distal tibia. While the traditional extensible intramedullary rod grows with the tibia, the outer sleeve with a thicker diameter will be far away from the middle and lower 1/3 of the distal tibia. At this time, because the traditional extensible intramedullary rod has a small inner core, it might cannot prevent re-fracture. Based on this, we improved the traditional extensible intramedullary rod, and placed the outer sleeve with a larger diameter at the distal end of the tibia, so that the intramedullary rod can always protect the distal end of the tibia. We propose a hypothesis that the new type of expandable intramedullary rod can better prevent re-fracture. The new type of expandable intramedullary rod is sleeved in the hollow sleeve tube body, and the top end of the flat end of the inner core is provided with an inner hexagon hole; The inner side of the opening end of the outer sleeve tube body is provided with an internal thread section for convenient insertion and replacement. The threaded tip is fixed in the distal tibial epiphysis. Traditional extensible intramedullary rod (designed by Fassier and Duval in 2001, also known as FD nail [17]). In 2011, Birke et al. [18] reported that two CPT patients with NF1 were treated with expandable intramedullary rods combined with bone grafting, and two of them were healed.

In this investigation, the union rate between the two groups was higher. The author analyzed that the reason might be related to the combined operation. Cox et al. [19] reported that there were complications of distal displacement of the intramedullary rod when the expandable intramedullary rod was used to treat osteogenesis imperfection. The traditional extensible intramedullary rod has a small inner core, which might not prevent re-fracture. Therefore, there is a risk of bending or breaking at the junction of the outer sleeve and the inner core of the intramedullary rod due to stress concentration. Based on the above reasons, the new type of intramedullary rod designed by the author, hoping to effectively fix the tibia, maintain the bone mechanical axis and prevent re-fracture. In this study, 10 (40%) patients in group A (traditional extensible intramedullary rod) had re-fracture, and 3 (13%) patients in group B (new extensible intramedullary rod group) had re-fracture. The incidence of re-fractures in children with CPT in group B was lower than that in group A, with a statistically significant difference (*P* = 0.036). This may be related to the thick outer sleeve protecting the middle and lower third of the tibia which is not easy to fracture. There was no significant difference between group A and group B in primary healing rate, ankle valgus, proximal tibial valgus, and unequal length of tibia.

With the growth of tibia, there is still a risk of displacement of the extensible intramedullary rod, and the displacement of the intramedullary rod is the most common complication. In 2016, Alzahrani et al. [20] reported that 4 children with CPT were treated with expandable intramedullary rods, with an average follow-up of 52.3 months, and 2 of them were displaced. In this investigation, the incidence of intramedullary rod displacement in group A was 80%, while that in group B was 61%, with no significant difference. The authors analyzed the reasons for the displacement of the new type of expandable intramedullary rod in children, which may be related to the less distal thread of the intramedullary rod screwing into the distal tibial epiphysis or the shallower distal thread. Therefore, the new extensible intramedullary rod needs to be further improved. For example, the distal thread of the intramedullary rod should be deepened to prevent the displacement of the intramedullary rod. When the intramedullary rod is placed, the distal thread should be as close to the ankle surface as possible, but should not enter the ankle. During the operation, the range of motion of the ankle should be checked to confirm that it does not affect the ankle movement. Besides, ankle arthrography was performed during the operation, and fluoroscopy confirmed that the distal thread of the intramedullary rod did not enter the ankle. However, the follow-up time of two groups is relatively short. We will continue to follow up the long-term results of traditional and new types of expandable intramedullary rods in the treatment of CPT.

## Conclusion

Compared with the traditional extended intramedullary rod combined operation, the new type of extended intramedullary rod combined operation has a lower incidence of re-fracture after CPT, but it still needs to be verified by large sample and multi-center research.

### Electronic supplementary material

Below is the link to the electronic supplementary material.

Supplementary Material 1

## Data Availability

The data are available on request. The data are not publicly available due to privacy restrictions. Data requests can be made to this email: meihaiboprofe@outlook.com.

## Abbreviations

CPT: Congenital pseudarthrosis of the tibia
NF1: Neurofibromatosis type 1
TLD: Tibial length discrepancy, PTV: proximal tibial valgus
