# Supplementary material for: Comparative study of different extendable intramedullary rods combined with surgery in the treatment of congenital pseudarthrosis of the tibia
Source: Orphanet J Rare Dis. 2024 May 21;19:208. doi: 10.1186/s13023-024-03202-0 (PMC11110408; doi:10.1186/s13023-024-03202-0)
Supplement: Supplementary file 1 — Supplementary Material 1 [file 13023_2024_3202_MOESM1_ESM.docx]

**1、 Exclusion criteria: patients with multiple tibial angulation (Why??).**

*Thank you for your question. In the early stages, we were concerned that children with tibial angular deformity had abnormal intramedullary rod core slippage, so they were not included.*

*2、***20 cases (80%) had new type of extensible intramedullary rod displacement. (this sentence is difficult to understand…what a new type of rod displacement ?? is it tip of the rod migration ??**

*Yes, it is tip of the rod migration.*

**3、If the plaster (???) fixation does not heal after re-fracture, surgery may be required.**

*If the* *cast immobilization does not achieve union, surgery may be required.*

**4、9 cases were associated with proximal tibial dysplasia (why using new telescopic rods with proximal defects ???)**

*Thank you. We believe that proximal tibial dysplasia is not a contraindication to the use of extendable intramedullary rods, and if the intramedullary rods cannot pass through the proximal tibia, we can perform proximal tibial closure osteotomy to correct alignment.*

**5、 Why proximal tibia dysplasia is not referred anymore ?**

*Thank you for your important suggestion. We have added the reference.*

*Cho TJ, Choi IH, Lee KS, et al. Proximal tibial lengthening by distraction osteogenesis in congenital pseudarthrosis of the tibia. J Pediatr Orthop. 2007; 27:915–920.*
